# The method based on ATR‐FTIR spectroscopy combined with feature variable selection for the boletus species and origins identification

**DOI:** 10.1002/fsn3.4369

**Published:** 2024-08-06

**Authors:** Zhiyi Ji, Honggao Liu, Jieqing Li, Yuanzhong Wang

**Affiliations:** ^1^ College of Resources and Environmental Yunnan Agricultural University Kunming China; ^2^ Institute of Medicinal Plants, Yunnan Academy of Agricultural Sciences Kunming China; ^3^ Yunnan Key Laboratory of Gastrodia and Fungi Symbiotic Biology Zhaotong University Zhaotong China

**Keywords:** feature variable selection, food safety, mid‐infrared spectroscopy, species identification, traceability, wild boletus

## Abstract

Wild boletus mushrooms, which are macrofungi of the phylum Basidiomycetes, are a nutritious and unique natural food that is widely enjoyed. Since boletus are consumed with problems of indistinguishable toxic and non‐toxic species and heavy metal enrichment, their species identification and traceability are crucial in ensuring quality and safety of consumption. In this study, the attenuated total reflection Fourier transform infrared (ATR‐FTIR) spectroscopy technique combined with three feature variable extraction methods, manual selection method, semi‐manual selection method, and algorithm method, were used to improve the accuracy and computational speed of the model identification, and the models were established for the identification of boletus species with an accuracy of up to 100% as well as for the identification of boletus origin with an accuracy of 86.36%. It was found that the best methods to improve the accuracy of the models were semi‐manual selection, manual selection and algorithmic selection in that order. This study can provide rapid and accurate species identification and origin traceability of wild boletus, and provide theoretical basis for the rational use of feature variable selection methods.

## Graphical abstract

The species and origins of wild boletus were identified by FT‐MIR combined with LIBSVM models and three feature variable selection methods. It was found that the best feature variable selection methods to improve the accuracy of the models were semi‐manual selection, manual selection, and algorithm selection in that order. The feature variable selection method should be chosen with care and the importance of a wider spectral range should be acknowledged.

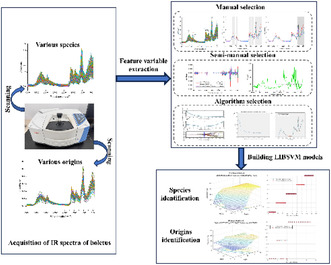

## 1 INTRODUCTION

Wild boletus mushrooms are delicious and widely consumed around the world because of their high‐protein and low‐fat properties and their antioxidant, antibacterial, antitumor, immunomodulatory, hepatoprotective, hypoglycemic, and antihypertensive activities (Liu, Sun, et al., 2023). Yunnan Province, the “Kingdom of Wild Mushrooms” in China, is the main production area of wild boletus. Local people have also shown a unique passion for boletus, not only can they taste this delicious flavor, but the boletus trade has also made a significant contribution to poverty alleviation and income generation in local rural areas (Fang et al., 2015).

The *Retiboletus fuscus*, *Lanmaoa asiatica*, *Boletus bainiugan*, *Rugiboletus extremiorientalis*, and *Butyriboletus roseoflavus* are five common available wild boletus edible mushrooms in the market. Among them, two species of boletus mushrooms contain toxins that cause diarrhea and psychedelia and require special and careful cooking when eating them. Thus it is necessary to identify them accurately. Locally, the *Lanmaoa asiatica* is known as “red jian shou qing” and “red onion,” names that derive from its distinctive onion‐like aroma and the rapid bluing of wounds caused by toxic substances. Although listed on the “Yunnan Common Poisonous Mushrooms (Poisonous Mushrooms) Version 2022” list jointly issued by the Kunming Institute of Botany of the Chinese Academy of Sciences and the Yunnan Key Laboratory of Fungal Diversity and Green Development, and the public has been strongly advised not to pick, purchase, process, or consume them (http://yn.people.com.cn/n2/2022/0424/c372456‐35238502.html), this flavorful boletus is still very popular. The *Butyriboletus roseoflavus* are commonly known as “white jian shou qing” or “white onion.” Because it appears to be non‐toxic and the wounds hardly turn blue or change color slowly after injury, people let their guard down when eating it and become poisoned. In terms of price, the “jian shou qing” boletus is also more expensive. The local people have a unique way of cooking these two poisonous mushrooms, which involves a lot of oil and prolonged cooking (Wang, Liu, et al., 2022). However, incidents of poisoning due to incomplete culinary maturation occur frequently. Therefore, boletus species identification is crucial to ensure the safety of consumers and prevent market fraud, especially for dried porcini slices that are more difficult to recognize from their appearance. In addition, different degrees of heavy metal enrichment in wild mushrooms due to different origin backgrounds make the safety of human consumption hazardous (Brzezicha‐Cirocka et al., 2016; Wang et al., 2017). Therefore, species identification and origin tracing are important to ensure the food safety of wild boletus.

In the identification of mushrooms as “poisonous” species, the public generally judge them by previous eating experience and basic characteristics (color, odor, and morphological observations), but it carries a high level of risk (Li, Zhang, et al., 2022). Currently, chemical, mass spectrometry, microscopy, DNA sequencing, and molecular labeling methods are commonly used (Liu et al., 2011; Parnmen et al., 2024; Sugawara et al., 2016; Wang, Gao, et al., 2022). Although the results are accurate, they usually involve exposure to chemical reagents and a long testing phase.

Infrared (IR) spectroscopy is commonly used for identification researches of various biological materials, whether plants, animals, or microorganisms (Bureau et al., 2019; Farouk et al., 2022; Hassoun et al., 2020; Ghooshkhaneh et al., 2023). In the food sector, IR spectroscopy combined with chemometrics is often used for fraud prevention and authenticity assurance. Shannon et al. (2022) used FTIR spectroscopy in conjunction with unsupervised Principal Component Analysis (PCA) method and supervised Orthogonal Partial Least Squares‐Discriminant Analysis (OPLS‐DA) model to successfully differentiate adulterated from unadulterated turmeric powder, with only 1 sample out of 228 samples being misclassified. The application of IR spectroscopy was affirmed by An et al. (2024) in the identification of *Fritillaria* species from various plant sources. Raw spectra, second‐order derivative (SD) processing, and two‐dimensional correlation spectroscopy (2DCOS) initially improved the resolution of FTIR spectra and successfully identified four types of wines (Wang, Hu, et al., 2022). Honey as a popular natural food is often the target of counterfeiting. Multivariate analysis of IR spectra was used to successfully identify honey from different plant and insect sources (Grabato et al., 2022). Compared with chemical methods, it has the advantages of being fast, convenient, green, and non‐destructive. Among them, the ATR‐FTIR technique is widely used for identification, content prediction, adulteration testing of solid and liquid materials due to its high signal‐to‐noise ratio, good reproducibility, and fast scanning speed. The ATR mode reduces the time required to process samples compared to the potassium bromide press method. ATR‐FTIR has been successfully applied for geographic traceability and species authentication. The geographical origin of oysters has a significant impact on their economic value and nutritional content, and ATR‐FTIR was used as a simple and green method to accurately differentiate between oyster products from eight geographical sources (Guo et al., 2023). A non‐invasive method was developed using ATR‐FTIR for the identification of carrots from two geographical indications and general production areas (Reale et al., 2023). Johnson et al. (2022) successfully identified five different plum varieties with 97%–100% accuracy using ATR‐FTIR technique combined with two different supervised models, SVM and PLS‐DA. In vitro antioxidant activity and colorimetric analysis combined with ATR‐FTIR spectral data could be used for the accurate identification of 25 ripe figs (Hssaini et al., 2021).

Due to the overlapping and complexity of IR spectroscopy signals leading to difficult interpretation of absorption peaks and computational overload, preprocessing and feature extraction methods are always performed (Kharbach et al., 2023). The effects of different preprocessing methods on the edible oil adulteration model were explored, and it was found that different preprocessing methods may produce redundancy, degrade, and improve the performance of the PLSR model, so it is crucial to choose the appropriate preprocessing method (Khodabakhshian et al., 2023). Generally, in previous studies, feature variable selection has led to better results from the model. A model for identifying the geographical origin of prickly pear by combining FT‐IR and Supervised Latent Dirichlet allocation (SLDA) with two feature bands at 1679–1618 and 1520–900 cm^−1^ was established by selecting the absorption peaks corresponding to the larger loading values of the principal components (Li et al., 2023). The OPLS‐DA model of geographical and temporal origins of crude palm oil samples established by determining the significant wavenumbers obtained good accuracy after elaborate spectrum analysis as well as band assignment (Rozali et al., 2023). The weak and overlapping peaks in the algal FT‐IR spectroscopy data subjected to second‐order derivative (SD) processing were clearer, thus increasing the available information, and some bands were used as a basis for identifying the seaweeds polysaccharide species (Gómez‐Ordóñez & Rupérez, 2011). However, there is no standard way of selecting feature variables for use in various discriminant models. Currently, common feature extraction methods are mainly categorized into manual selection method, semi‐manual selection method, and algorithm selection method. The manual selection method directly selects feature bands by observing and analyzing spectral absorption peaks. Semi‐manual selection method, which evaluates the contribution degree of each variable and selects the variable with more important contribution for subsequent modeling, has both subjective selection part and combines objective analysis of spectrum data. The algorithm selection method relies entirely on the modeling of feature extraction algorithms with different principles.

The feature band selection methods are commonly used to remove the redundant bands without important information and select the bands with greater contribution rate for modeling, which improves the computational speed and model accuracy (Ghooshkhaneh et al., 2023; Kapoor et al., 2022). However, it also has the risk of losing the important information in the spectral bands if an unsuitable feature extraction method is selected (Shi et al., 2024). In this study, the classification models of boletus species and geographic origin based on different feature extraction methods will be established, the effect of feature extraction methods on the accuracy of the classification model will be studied, and reasonable suggestions for the application of feature extraction methods will be made.

## 2 MATERIALS AND METHODS

### 2.1 Material collection and preparation

A total of 375 samples (a fruiting body as a sample) of 5 wild boletus species, namely, *Retiboletus fuscus*, *Lanmaoa asiatica*, *Boletus bainiugan*, *Rugiboletus extremiorientalis*, and *Butyriboletus roseoflavus*, were collected from Kunming, Chuxiong, and Yuxi cities in the central region of Yunnan Province, China. The identity of the samples was confirmed through taxonomic identification by all authors based on the “Annals of Fungi of China: Boletaceae” (Zang, 2006) and “Common edible and poisonous mushrooms of southwestern China” (Yang et al., 2021) combined with the natural growth environment and fruiting body morphology of the collected samples. The boletus samples were processed after collection and transportation to the laboratory within 12 h. Used a soft brush to brush off the dust and soil on the surface of boletus, cleaned the surface as far as possible without damage, dried the slices at room temperature. These samples were then dried in a 101A‐1 electric blast drying oven (Chongming Experimental Instrument Factory, Shanghai) until constant weight, and then cooled them down and crushed the dried slices of *Boletus edulis* in FW‐100 high‐speed pulverizer (Huaxin Instrument Factory, Tianjin) and passed through a 100‐mesh sieve. The powder was put into the ziplock bags and stored in a dry and lightproof environment. The sample information is shown in Table 1.

**TABLE 1:** Sample information of five species of boletus.

Species	Origin and quantity			
	Kunming city	Yuxi city	Chuxiong city	All
*Retiboletus fuscus*	9	26	26	61
*Lanmaoa asiatica*	30	18	33	81
*Boletus bainiugan*	15	49	24	88
*Rugiboletus extremiorientalis*	22	16	29	67
*Butyriboletus roseoflavus*	7	37	34	78
All				375

### 2.2 ATR‐FTIR acquisition and exploratory analysis

An appropriate amount of boletus powder (2 ± 0.2 mg) was placed on the surface of diamond crystal (2 mm in diameter) of ATR accessory of FT‐MIR spectrometer (PerkinElmer, USA), and the sample was scanned by rotating the pressure lever to press the sample. The scanning range was 4000–400 cm^−1^ with a resolution of 4 cm^−1^, the number of scans was 64, and the scans of each sample were repeated three times to average the spectra. Background spectra were obtained every half hour by scanning the blank sample stage. Background spectrum scanning is performed before sample scanning in order to maximize the measurement error caused by instrument performance and water in the environment to obtain the spectrum of the sample itself. The temperature of the chamber was kept constant and relatively dry during the scanning process. Before scanning the next sample, the accessory table was wiped with alcohol.

After obtaining the average spectra of each sample, the spectral data of the different origins and species of samples were individually subjected to PCA – an unsupervised machine learning approach for exploratory analysis and data visualization, by which high‐dimensional data are downscaled and mapped into a low‐dimensional space to show the variability and features among the data.

### 2.3 Feature variable selection

Too many features increase model complexity and overfitting, and too few features lead to underfitting of the model. IR spectroscopy is often affected by noise, so some preprocessing methods are often used to reduce noise in spectroscopy. However, for different experimental materials, the preprocessing methods chosen based on previous experience do not necessarily have only positive effects, but also result in redundancy and decreased model accuracy (Mishra et al., 2021). Here, in order to better investigate the influence of the choice of feature variables, the influence of the preprocessing methods is not discussed and only the raw and unpreprocessed spectra are used for modeling.

#### 2.3.1 Manual selection method

Feature variable selection by observing the feature peaks of IR spectra and resolving the spectral information is a simple, fast, and effective method. Usually, there are feature wave number selection and feature band selection. As a common empirical selection method, it is often possible to compare spectral images to obtain the chemical bonding vibration regions of the functional groups representing the variability of a particular class of compounds or to remove regions without significant signal‐to‐noise ratios (De Freitas et al., 2021; Rozali et al., 2023). Fingerprint regions are overlapping regions of various chemical bonding vibrations that exhibit less information about functional groups and are not suitable for quantitative studies (Lv et al., 2020). However, it is sensitive to variations and is the best band for distinguishing subtle differences between molecules of different samples and is suitable for qualitative studies, especially for samples with extremely similar chemical information. Therefore, the feature wavenumbers, feature bands and fingerprint regions were manually selected as input variables by directly observing the shapes of the feature peaks of the original spectra and performing band assignments without the chemometric methods.

#### 2.3.2 Semi‐manual selection method

##### Variance spectrum selection

The average spectra of Boletus species and origins were analyzed by variance analysis in TQ Analysis software to obtain the deviation range for each average spectrum to obtain interclass distances. When performing the variance analysis, select the second‐order (SD) derivative treatment in the “Data Format” to separate the weak absorption peaks and the overlapping absorption peaks, which was used to enhance the spectral resolution and facilitate the manual selection of bands with large differences as feature variables (Wang, Hu, et al., 2022; Zheng et al., 2023). The feature peaks of the spectra were not shifted due to the derivative processing, but only made the feature peaks more obvious, improved the spectral resolution, and also eliminated the spectral baseline drift. The selected SD treatment was not applied as a pre‐processing method because the data set after the SD treatment was not brought into the classification model. The aim was to enhance the raw spectral variability for feature bands selection in this way.

##### Variable importance projection (VIP) selection

The VIP score reflects the importance of independent variables for the degree of model fit. List of VIP scores for each variable obtained by PLS analysis through SIMCA 14.1 software. In order to identify the spectral regions with the strongest discriminatory power and to explore the ability of the size of the VIP score to explain the labelling of the wavelength points, the variables with VIP score >1 and VIP score >1.5 were selected as feature variables, respectively (Liu et al., 2020; Márquez et al., 2016).

#### 2.3.3 Algorithm selection method

Algorithm selection method based on different evaluation criteria can quickly remove a large number of irrelevant samples. Competitive adaptive reweighted sampling (CARS) follows the principle of “survival of the fittest” proposed by Darwin's theory of evolution, and obtains N subsets by running N samples, and selects the subset with the lowest cross‐validation root mean square error (RMSECV) value as the most optimal subset (Li et al., 2009). Successive projections algorithm (SPA) is an unsupervised feature selection method that aims to select the wavelengths with the least redundancy in the spectral information in order to solve the problem of covariance, and the operation is simple and fast (Araújo et al., 2001). The projection operation of the candidate subset is first selected and the candidate subset is evaluated according to the Predicted residual error sum of square (PRESS), and the minimum number of variable subsets can be obtained by eliminating the variables in the end.

Compared to the three methods, the manual selection method is simpler and faster as it reduces the process of handling spectral data and modeling. However, some machine learning methods to help obtain the feature bands seem to be more scientific and convincing. Therefore, further use of classification models is needed to determine the performance of different feature selection methods.

### 2.4 Support vector machine (LIBSVM)

SVM is a supervised learning algorithm that maps linearly indivisible in low‐dimensional space to linearly divisible in high‐dimensional space. LIBSVM is a support vector machine library that implements multivariate classification (Abdiansah & Wardoyo, 2015). The model uses the radial basis function (RBF) as the kernel function. In this study, the classification performance of LIBSVM models was evaluated in terms of the value of the penalty factor *C*, the value of the kernel function radius *g*, and the accuracies of the training and test sets for different models. The Kennard‐Stone (K‐S) algorithm was first utilized to divide the training and test sets, where the training set accounts for 75% of the total sample size and the testing set accounts for 25% of the total sample size. The input data were spectral data, and the output data were species and origin categories of boletus. The optimal values of *C* and *g* were selected through the grid search method, and a sophisticated selection was made after going through a rough selection. The model fits well and runs fast when the value of *C* ranges from [2^−2^, 2^4^] while *g* ranges from [2^−4^, 2^4^]. Accuracy is the proportion of correctly classified predictions out of all results, see Equation (1). The closer the Acc value is to 100%, the superior the model classification performance.

(1) $$\text{Accuracy}=\frac{\mathrm{TP}+\mathrm{TN}}{\mathrm{TP}+\mathrm{TN}+\mathrm{FP}+\mathrm{FN}}$$

where TP, TN, FP, and FN are true positive, true negative, false positive, and false negative samples respectively.

OMNIC software was used for synthesizing the mean spectra, SIMCA 14.1 software was used to convert the spectral profiles into the spectral data matrix, Origin 2021 software for plotting, and MATLAB R2023a software for modeling.

## 3 RESULTS AND DISCUSSION

### 3.1 Spectral analysis

**FIGURE 1:**
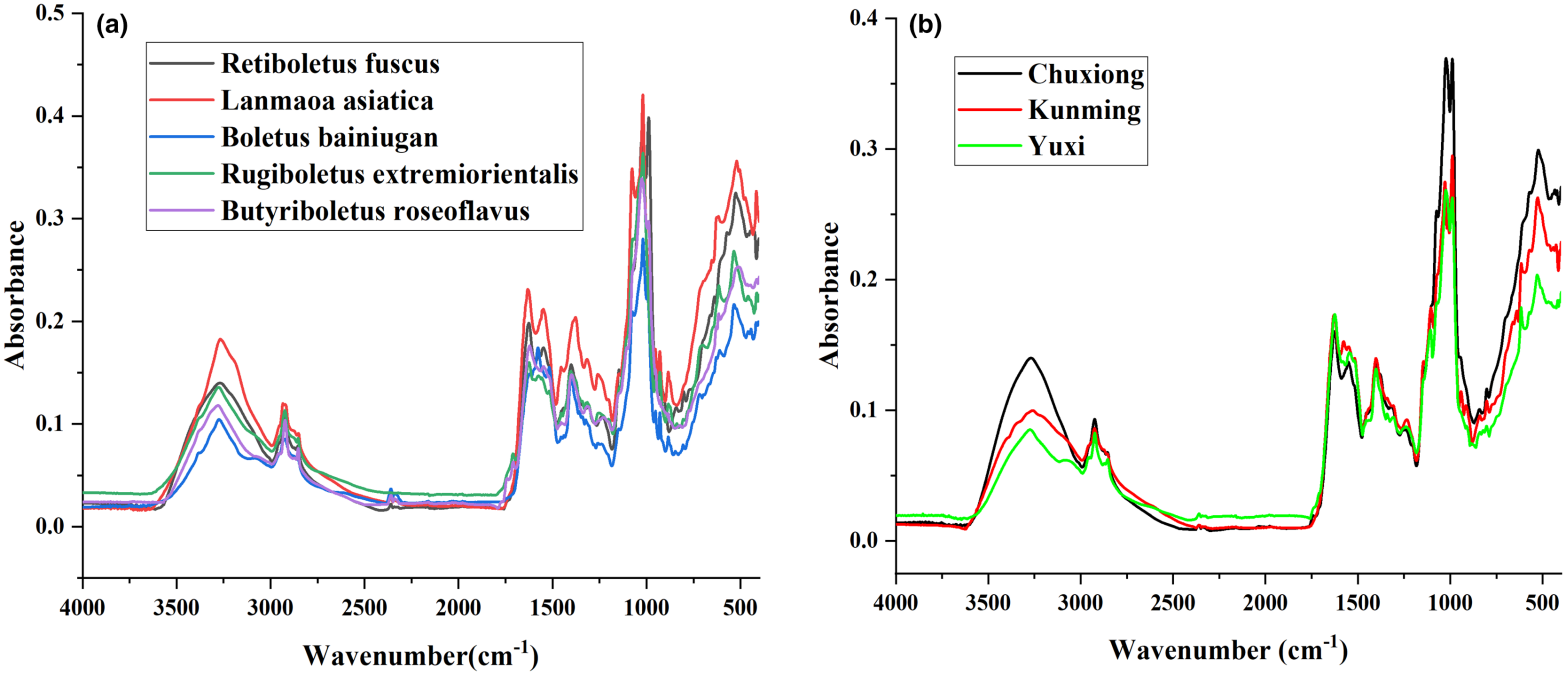
Average ATR‐FTIR spectra of boletus from different species (a) and different origins (b).

The raw average MIR spectra of boletus samples of different species and different origins are shown in Figure 1a,b, respectively. The peak shapes of all the boletus spectra are basically the same, with obvious overlapping. The samples of different origins were part of the samples of different species of *Boletus edulis*. The 10 distinct absorption peaks present in all of them were 3280, 2920, 2850, 1620, 1550, 1390, 1320, 1230, 1020, and 520 cm^−1^. On average, each spectrum contained 7468 variables. 3280 cm ^−1^ near the broad wavenumber region is associated with the O–H stretching vibrations of proteins and fatty acids (De Freitas et al., 2021; Liu, Sun, et al., 2023). The two narrow absorption peaks at 2850, 2920 cm^−1^ are associated with the symmetric and asymmetric stretching vibrations of the aliphatic –CH_3_– and –CH_2_– groups (Cervantes‐Paz et al., 2022; De Freitas et al., 2021; Liu, Xiao, et al., 2023; Lv et al., 2024). The –OH stretching vibrations of the polysaccharide feature information are associated with 1900–900 cm^−1^ related (Baca‐Bocanegra et al., 2022). The 2000–1500 cm^−1^ is a double‐bond vibrational region, and the absorption bands near 1620 and 1550 cm^−1^ are attributed to the stretching vibrations of the C=O bond to the C=C bond of the triglyceride O–C=O and polyunsaturated fatty acid CH=CH moieties (De Freitas et al., 2021; Rozali et al., 2023). The vicinity of 1390 cm^−1^ is associated with the C=O moiety in aldehydes, esters, and ketones (De Freitas et al., 2021). The absorption peak at 1320 cm^−1^ is associated with the C–H bending vibration of cellulose (Liu, Xiao, et al., 2023; Ma et al., 2018; Rozali et al., 2023). The small absorption peak at 1230 cm^−1^ is associated with the C–N bending vibration of the amide moiety of proteins (De Freitas et al., 2021; Merriman et al., 2022), and the high absorption peak near 1020 cm^−1^ is attributed to the telescopic vibration of the primary and secondary alcohol C–O bonds of glucose in starch (Chen et al., 2012; Shannon et al., 2022). The absorption peak at 520 cm^−1^ is related to the vibration of the alkyl rings of pyran compounds skeleton in glycosides (Liu, Xiao, et al., 2023).

### 3.2 Exploratory analysis

**FIGURE 2:**
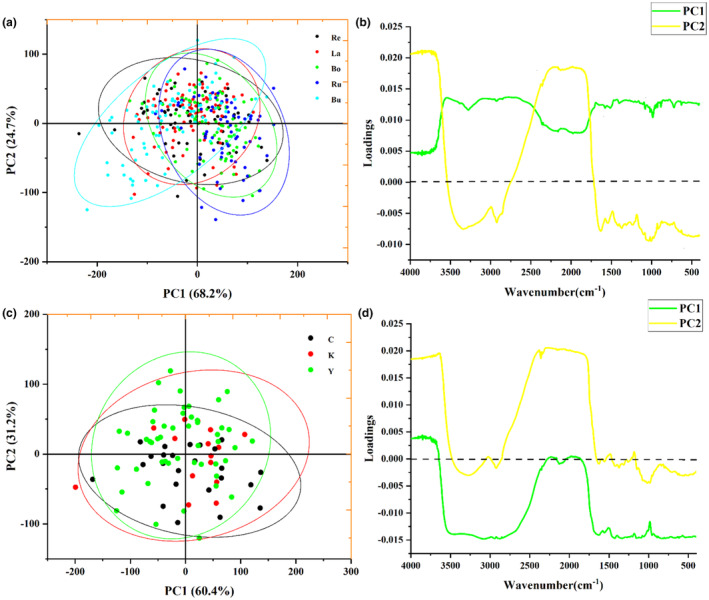
The score plot (a) and loading plot (b) of PCA of different species of *Boletus edulis*; the score plot (c) and loading plot (d) of PCA of different origins of *B. edulis*.

Figure 1a,b show the average spectra of boletus from various species and origins, and it is evident that the boletus samples all have similar peak shapes and positions, which indicates that these mushroom substrates contain comparable chemical compositions. According to the height of the peaks, *Lanmaoa asiatica* have higher absorption peaks compared with other boletus species, and the *Boletus bainiugan* from Chuxiong have greater differences with those from Kunming and Yuxi. According to Lambert's law, this is mainly due to the different content of chemosynthetic components. Figure 2 shows the PCA score plots and loading plots for different species and origins of *Boletus edulis*. Samples exceeding the 95% confidence interval were identified as outliers. Even though removing outliers could make the model results more favorable, retaining them is more representative of the actual situation and encompasses a more diverse range of real samples. It is the reason that outlier removal is not performed. In Figure 2a, all five boletus species have a better clustering situation, but there is some overlap and they cannot be well distinguished. And the same is true for white boletus from three different origins. Among them, principal component 1 (PC1) and principal component 2 (PC2) of different species of boletus explained a total of 92.9% of the variance contribution, and different origins of boletus explained a total of 91.6%. PCA could not reveal the significant separation of boletus species and origins. However, the clustering of the score plots demonstrates to the classification potential of samples of different species and origins of boletus, and further analysis is needed.

### 3.3 Evaluation of the LIBSVM model

#### 3.3.1 Manual selection

Tables 2 and 3 show the number of factors, the optimal *C* and *g* values, and the accuracies of the training set and test set for each LIBSVM model based on different feature variable extraction methods, respectively. Five variables each near the 10 feature peaks at 3280, 2920, 2850, 1620, 1550, 1390, 1320, 1230, 1020, and 520 cm^−1^, totaling 50 variables, were selected as feature variables. This feature extraction method resulted in a decrease in the training accuracy for classification of both different species and origins of boletus mushrooms, while the test accuracy for different species increased slightly and the test accuracy for the origins classification model remained unchanged. This did not significantly improve model performance, but the number of variables to perform the modeling was only about 1/150 of the full‐spectrum, greatly reducing redundant variables and saving time costs.

**TABLE 2:** Results of the LIBSVM model for species identification of boletus based on different variable selection methods.

Spectral variable selection	Factors	Best *C*	Best *g*	ACC	
				Train	Test
RAW	7468	9.27 × 10^4^	1.35 × 10^−6^	96.09%	97.87%
Manual wavenumbers	50	1.02 × 10^3^	1.56 × 10^−2^	91.10%	98.94%
Manual wave bands	3309	5.79 × 10^3^	3.45 × 10^−4^	96.44%	100%
Fingerprint region	1246	8.19 × 10^3^	6.10 × 10^−5^	96.44%	98.94%
SD	4121	6.55 × 10^4^	3.81 × 10^−6^	97.15%	100%
VIP (>1)	2122	1.16 × 10^4^	1.53 × 10^−5^	96.09%	100%
VIP (>1.5)	549	4.63 × 10^4^	1.22 × 10^−4^	97.15%	98.94%
CARS	68	5.24 × 10^5^	2.76 × 10^−3^	84.70%	95.74%
SPA	59	1.31 × 10^5^	1.73 × 10^−4^	96.44%	100%

**TABLE 3:** Results of the LIBSVM model for origin identification of *Boletus bainiugan* based on different variable selection methods.

Spectral variable selection	Factors	Best *C*	Best *g*	ACC	
				Train	Test
RAW	7468	2.32 × 10^4^	1.08 × 10^−5^	87.88%	72.73%
Manual wavenumbers	50	3.71 × 10^5^	1.95 × 10^−3^	72.73%	72.73%
Manual wave bands	3100	5.24 × 10^5^	7.63 × 10^−6^	81.82%	81.82%
Fingerprint region	1246	4.10 × 10^3^	6.91 × 10^−4^	77.27%	77.27%
SD	4259	2.32 × 10^4^	3.02 × 10^−5^	89.39%	86.36%
VIP (>1)	2950	3.71 × 10^5^	2.70 × 10^−6^	89.40%	72.73%
VIP (>1.5)	288	1.02 × 10^3^	1.10 × 10^−2^	74.24%	68.18%
CARS	73	1.05 × 10^6^	4.88 × 10^−4^	78.79%	72.73%
SPA	17	1.64 × 10^4^	7.81 × 10^−3^	87.88%	68.18%

**FIGURE 3:**
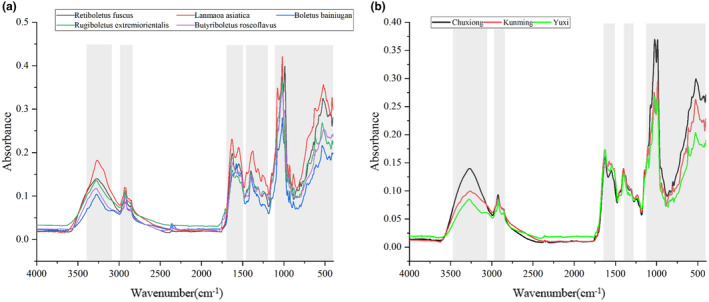
The feature bands were selected by manual selection of different species (a) and different origins (b) of boletus. The gray zone indicated the region of interest.

By comparing the average spectra of different species of boletus, as Figure 3a, it was found that the spectral trends of the five species of the mushrooms were more or less the same, but with more obvious differences at some bands, which reflected the differences in the internal chemical composition and content of different species of *Boletus edulis*. In particular, *Lanmaoa asiatica* was more different from the other four species. These spectral bands were marked with gray shading as feature variables, which were 3390–3100, 2980–2830, 1680–1480, 1450–1190, and 1090–400 cm^−1^, respectively. Similarly, Figure 3b demonstrates that among different origins of *Boletus bainiugan*. Boletus from Chuxiong city had more significant differences with the other two origins differed significantly. A total of 3100 variables of 3460–3050, 2950–2840, 1630–1500, 1390–1270, and 1120–400 cm^−1^ were selected as the feature variables, as shown in Tables 2 and 3. The manual selection of feature bands achieved better results in improving the classification accuracy of the model. In the boletus species classification model, the training set accuracy was slightly improved and the test set accuracy reached 100%. The test set classification accuracy increased in the boletus origin classification model even though the training set classification accuracy decreased.

The IR spectral fingerprint region of *Boletus edulis* at 1200–400 cm^−1^ is rich in chemical information and sensitive to changes. In the spectral images of different species of *Boletus edulis* and different origins of *Boletus edulis*, the absorption peaks in the fingerprint region have large differences, which can be used as a basis for classification. A LIBSVM model was built using only this region, and the accuracy of the test set increased in all cases compared to the original spectral model, but the accuracy of the training set decreased in all cases compared to the manually selected model with gray‐shaded feature bands. This may be due to the fact that the selection of the fingerprint region removes redundant information compared to the original spectra but removes some more effective information compared to the feature bands.

#### 3.3.2 Semi‐manual selection

**FIGURE 4:**
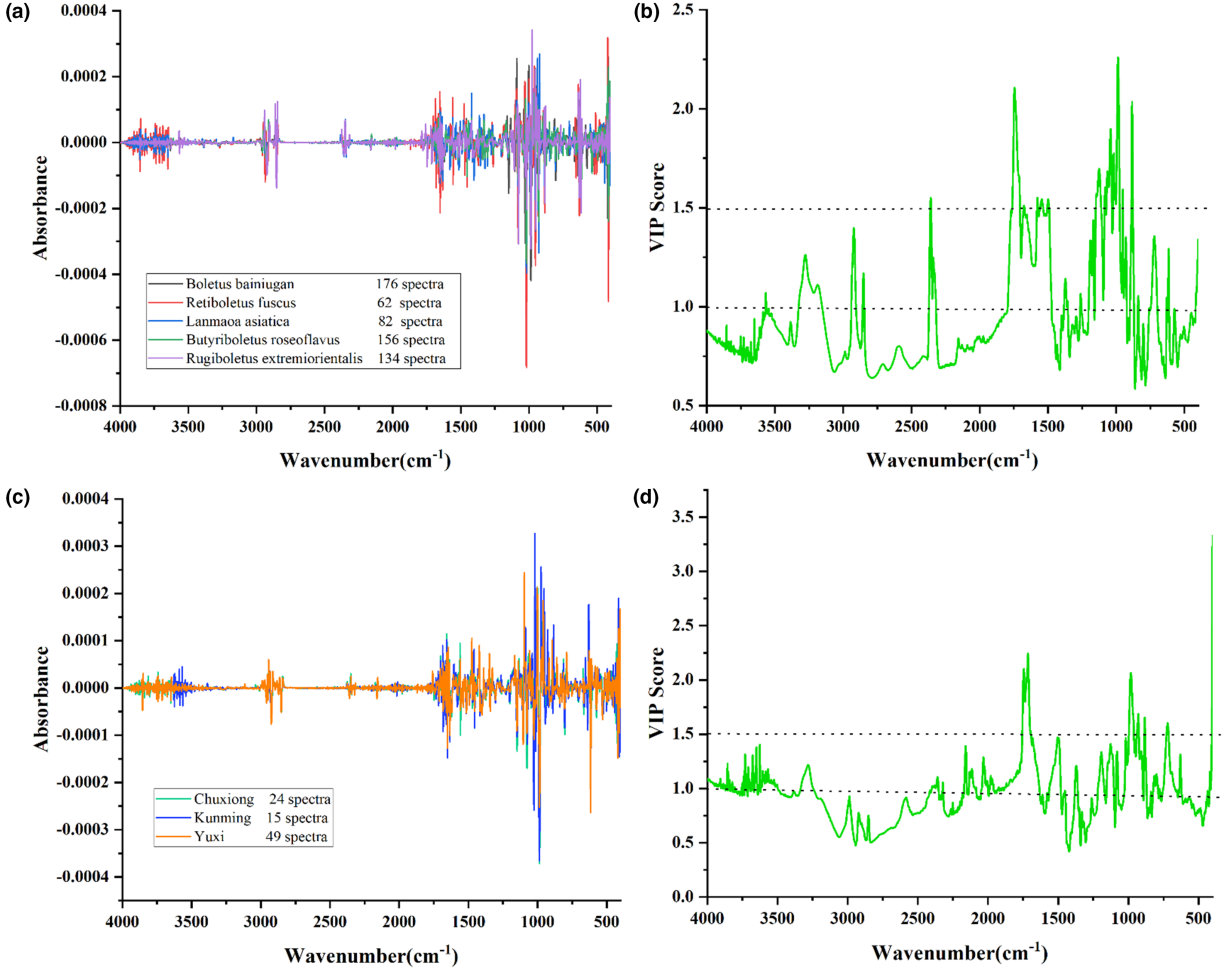
Semi‐manual selection process for feature variables. (a) and (b) are boletus species identification model, (c) and (d) are boletus origin identification model. (a) and (c) show the results of the variance spectra after SD treatment. (b) and (d) show the distribution of different VIP values for 7468 variables of the MIR of *Boletus edulis*.

The semi‐manual extraction method was used to determine the characteristic difference bands and the number of difference‐contributing waves by variance spectra and VIP score, respectively. The results of the variance spectra show interclass differences. According to Figure 4a, the deviation ranges of *Retiboletus fuscus*, *Lanmaoa asiatica*, *Boletus bainiugan*, *Rugiboletus extremiorientalis*, and *Butyriboletus roseoflavus* were 82, 159, 176, 134, and 62 spectra, respectively. The deviation ranges from three different origins of *Boletus bainiugan* were 24 spectra, 15 spectra, and 49 spectra, respectively, as shown in Figure 4c. In comparison with Figure 1a,b, the deviation range of *Boletus bainiugan* was larger, and *Boletus bainiugan* from Yuxi had a greater difference from the other two origins, which was different from the results obtained by the direct observation method. The spectral resolution could be enhanced by SD processing. Bands with significant differences were selected as feature bands in the SD processing spectral profiles, and the remaining highly overlapping bands were removed as non‐feature bands. After deleting about 3000 non‐feature variables, the performance of the SD‐LIBSVM models built separately all obtained the best classification performance. According to Figures 3 and 4, the feature bands selected in the SD derivative spectra were compared with the manual wave bands in the original spectra, and the band at 4000–3500 cm^−1^ was selected, while the band at 3600–3000 cm^−1^ was not selected. This is due to the fact that SD processing excludes the effect of baseline drift making the overlapping severe signal enhancement. 2800–1700 cm^−1^ has no significant fluctuation and is therefore excluded. In addition, the signal variations in the fingerprint region are extremely sensitive. In the raw spectra, regions with significant overlap were not selected, whereas in the SD derivative spectra, the region at the 1700–400 cm^−1^ band was selected in its entirety because of the very significant signal variations exhibited in this region. The accuracy of the established LIBSVM models proved that the feature bands selected by the SD method performed better than direct manual selection on the original spectra for species and origins identification of *Boletus edulis* (Tables 2 and 3). In the species identification model, the accuracy of the train and test sets changed from 96.44% and 100.00% to 97.15% and 100.00%, respectively, and the accuracy of the origins identification model increased from 81.82% and 81.82% to 89.39% and 86.36%. The higher model accuracy of the SD method compared to direct manual extraction of feature bands is due to the improved feature resolution. The selection of VIP score is shown in Figure 4b,d. In the boletus species identification model, 2122 and 549 variables with VIP value >1 and VIP value >1.5 were selected by VIP extraction method, and the accuracy of the test set was improved by 2.13% and 1.07%, respectively, compared with the original spectra. However, the accuracy of the feature variable models with VIP values >1.5 was lower than the accuracy of VIP values >1. Although the variables with VIP value >1.5 are more capable of interpreting labels, they also lose a lot of important information. In the boletus origin identification model, the accuracy of the training set of the model with VIP value >1 slightly increased but the accuracy of the test set remained unchanged, and the accuracy of both the training and test sets of the model with VIP value >1.5 decreased. Similarly, the number of variables with VIP values >1.5 was only 288, removing too much valid information compared to the variables with VIP values >1.

#### 3.3.3 Algorithm selection

The boletes species identification models were extracted and LIBSVM modeled with 68 and 59 variables using the CARS and SPA algorithms, respectively. Compared with the original spectra, the accuracy of both the training and test sets of the CARS‐LIBSVM model decreased, while the accuracy of the SPA‐LIBSVM model increased. Whereas, the boletus origin identification models with variables selected by CARS and SPA formed a decrease in the training set accuracy and a decrease in the test set accuracy, respectively. From the process of variable selection in Figures S1 and S2, the results of variables extracted by the two algorithms are quite different due to the different principles and methods they are based on. The region selected by the CARS algorithm was only concentrated at 4000–3000 cm^−1^, while the variables selected by the SPA algorithm were much closer to the spectral bands manually selected with obvious differences.

### 3.4 Comparison of the feature selection methods

Figures S3 and S4 display the classification results of LIBSVM models of the test and training sets of boletus species and origins under different feature variable selection methods. In the boletus mushroom species identification model, the classification accuracy of the model after different feature extraction methods was improved to some extent, except for the CARS‐SNV model, which showed a decrease in classification performance. In the boletus origin identification model, the manually extracted feature band, fingerprint region, and SD methods obtained better results. In general, the manual and semi‐manual extraction of feature bands was more effective than the algorithmic extraction. Unpreprocessed FT‐IR spectra have significant noise and baseline drift, which affects the algorithmic extraction of feature: The wavelets caused by the noise are recognized as feature wavenumbers. The advantage of high sensitivity of algorithmic extraction also happens to be a disadvantage of the method. Based on the optimal preprocessing in the algorithm to extract feature variables can greatly improve the accuracy of model recognition, but the choice of optimal preprocessing undoubtedly increases the workload. Different algorithms are designed based on different principles, and different algorithms are applicable to different samples, which leads to poor generalization ability and robustness of the algorithm to extract feature variables. Manual selection of wavenumber can be more intuitive to select the feature wave that causes differences, but it has a strong sense of subjectivity. Meanwhile, in the case of small sample sizes, feature variable extraction is used with caution, and full‐spectrum datasets are used as much as possible to build discriminative models. This may be due to the fact that small sample datasets are not characterized significantly and feature extraction can lead to the loss of important feature information. It may also be due to the fact that the samples are all from extremely geographically similar origins. Geographic traceability models developed using small regional samples with similar features can be applied to origins identification of the same species on a broader regional scale, enhancing the applicability and extensiveness of the models.

In our past work, the species and origins of *Boletus edulis* have been identified using IR spectroscopy and chemometric methods, both with an accuracy of more than 95% (Liu, Liu, et al., 2023; Wang et al., 2021). However, related studies were based on the validation and comparison of different chemometric methods. In addition, the species and origins identification models based on manual selection of feature wave bands and semi‐manual selection of SD processing constructed in this study had 100% and 86.36% accuracy, respectively. However, the classification model constructed in this study could only identify boletus from the five species and three origins mentioned in the researches, and if samples of *Boletus edulis* from different regions or even different countries were used, it is necessary to re‐collect the spectral data of these samples to train and validate the model, and then subsequently use it for the discrimination of new samples. This is one of the limitations of this study.

The use of feature selection methods in qualitative and quantitative of IR spectroscopy studies yielded similar results compared to previous studies. Fermented and unfermented cocoa beans were identified using visible (VIR) and near‐infrared (NIR) spectroscopy. The average spectral difference feature peaks of the two cocoa beans were resolved by manual selection method, and three feature bands were selected for PLS‐DA and LDA modeling respectively, which resulted in a successful identification model with a smaller and more accurate range (Pinto et al., 2024). For another study, the prediction of total volatile basic nitrogen (TVB‐N) content in fish using NIR spectra and Raman spectra using data concatenation of the two raw spectra (low‐level data fusion, LLDF) and data concatenation of the two spectral feature bands selected by the VIP value (mid‐level data fusion, MLDF) resulted in higher prediction accuracies as compared to the two algorithmic extraction methods, CARS and iteratively retains informative variables (IRIV). The reasons for this were that the raw spectra have more comprehensive information and the VIP value method can extract more feature peaks associated with proteins, while changes in TVB‐N content are associated with protein denaturation (Guo et al., 2024). For the treatment of predicting polysaccharide content in wine, the region of 1900–900 cm^−1^, which was highly correlated with polysaccharide features, was manually selected for subsequent modeling (Baca‐Bocanegra et al., 2022). Of course, in many studies, such as the study of the classification of different processed products of aromatic herbs, algorithms extracting feature variables also often achieved better modeling results compared to the original spectra (Shi et al., 2024). Therefore, regardless of the method used, the key to feature band selection is whether the extracted bands are relevant to the purpose of the study. Manual and semi‐manual selection of feature bands should be done in conjunction with the extent to which the variables explain the identification and prediction.

## 4 CONCLUSION

Each model had a high accuracy, and the accuracy of the test set of the model for boletus species and origins identification within adjacent regions is 95.74%–100.00% and 68.18%–81.82%, respectively, indicating that IR spectroscopy was suitable for the application of boletus species and origin identification. Through three different feature extraction methods: manual selection by directly selecting feature regions by observing spectral images, semi‐manual selection after improving spectral resolution and calculating VIP score, and algorithm selection by directly relying on algorithms, it was found that, in general, manual and semi‐manual selection of the feature variable models worked better than selection through algorithms, and that the selection of the feature variables should not be completely dependent on algorithms. Although too many redundant variables in the full spectrum could lead to slow computation of the classification model and could not be better applied for fast market identification. However, inappropriate feature variable selection methods can cause the model identification accuracy to decrease, which is not in line with the purpose of building a better model, especially for some special requirements, such as toxic and non‐toxic boletus species identification models where 100% classification accuracy is desired. Therefore, when performing feature variable extraction, it is necessary to choose the appropriate way according to different sample sizes and spectral characteristics, and avoid choosing based on past experience. This also provides some ideas for other researchers to consider the way of feature variable selection.

## AUTHOR CONTRIBUTIONS

**Zhiyi Ji:** Conceptualization (equal); data curation (equal); formal analysis (equal); methodology (equal); writing – original draft (equal). **Honggao Liu:** Funding acquisition (equal); project administration (equal); supervision (equal); validation (equal). **Jieqing Li:** Investigation (equal); resources (equal); software (equal); supervision (equal); writing – review and editing (equal). **Yuanzhong Wang:** Resources (equal); software (equal); supervision (equal); visualization (equal); writing – review and editing (equal).

## CONFLICT OF INTEREST STATEMENT

The authors declare that they have no known competing financial interests or personal relationships that could have appeared to influence the work reported in this paper.

## Supporting information


Figure S1.–S4.

## Data Availability

Data available on request from the authors: the data that support the findings of this study are available from the corresponding author [Jieqing Li; Yuanzhong Wang] upon reasonable request.
