# Supplementary material for: The method based on ATR‐FTIR spectroscopy combined with feature variable selection for the boletus species and origins identification
Source: Food Sci Nutr. 2024 Aug 6;12(10):7696–707. doi: 10.1002/fsn3.4369 (PMC11521652; doi:10.1002/fsn3.4369)
Supplement: Supplementary file 1 — Figure S1.–S4. [file FSN3-12-7696-s001.docx]

**Supplementary Materials**

**Fig.S1.** CARS model selection process for feature variables.

**Fig.S2.** SPA model selection process for feature variables.

**Fig.S3.** Classification results of LIBSVM models of the test and training sets of boletus species under different feature variable selection methods.

**Fig.S4.** Classification results of LIBSVM models of the test and training sets of boletus origins under different feature variable selection methods.

**
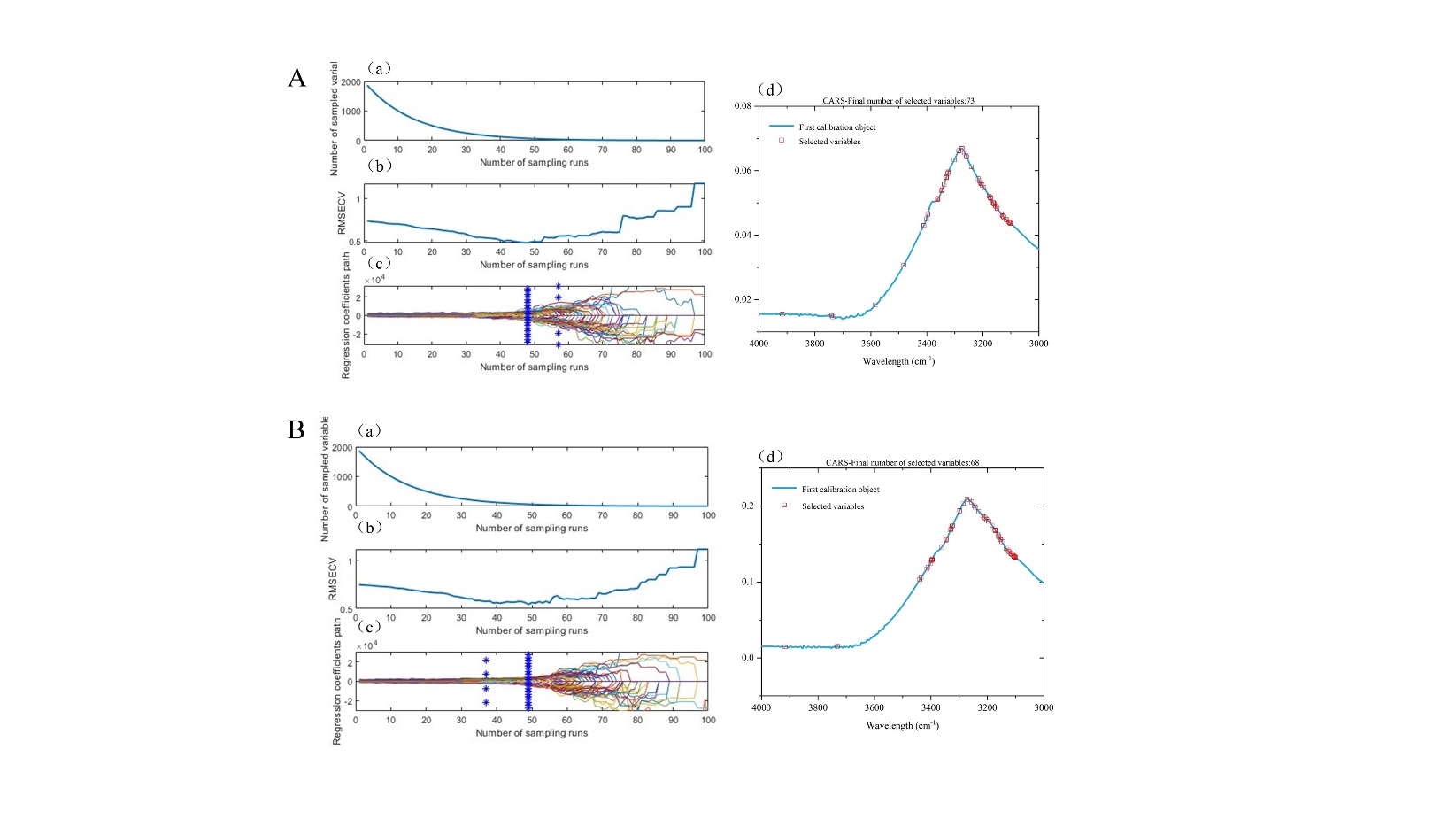
**

**Fig.S1.** CARS model selection process for feature variables. A: Boletus species identification model, B: Boletus origin identification model. Where (a) shows the decrease in the number of variables selected as the number of runs increases. (b) shows the change in RMSECV due to the increase in the number of runs, where the RMSECV value of the model decreases and then increases as the number of runs increases while the number of variables decreases. (c) shows the regression coefficients path as the number of runs increases. (d) shows the results of the CARS model selection.


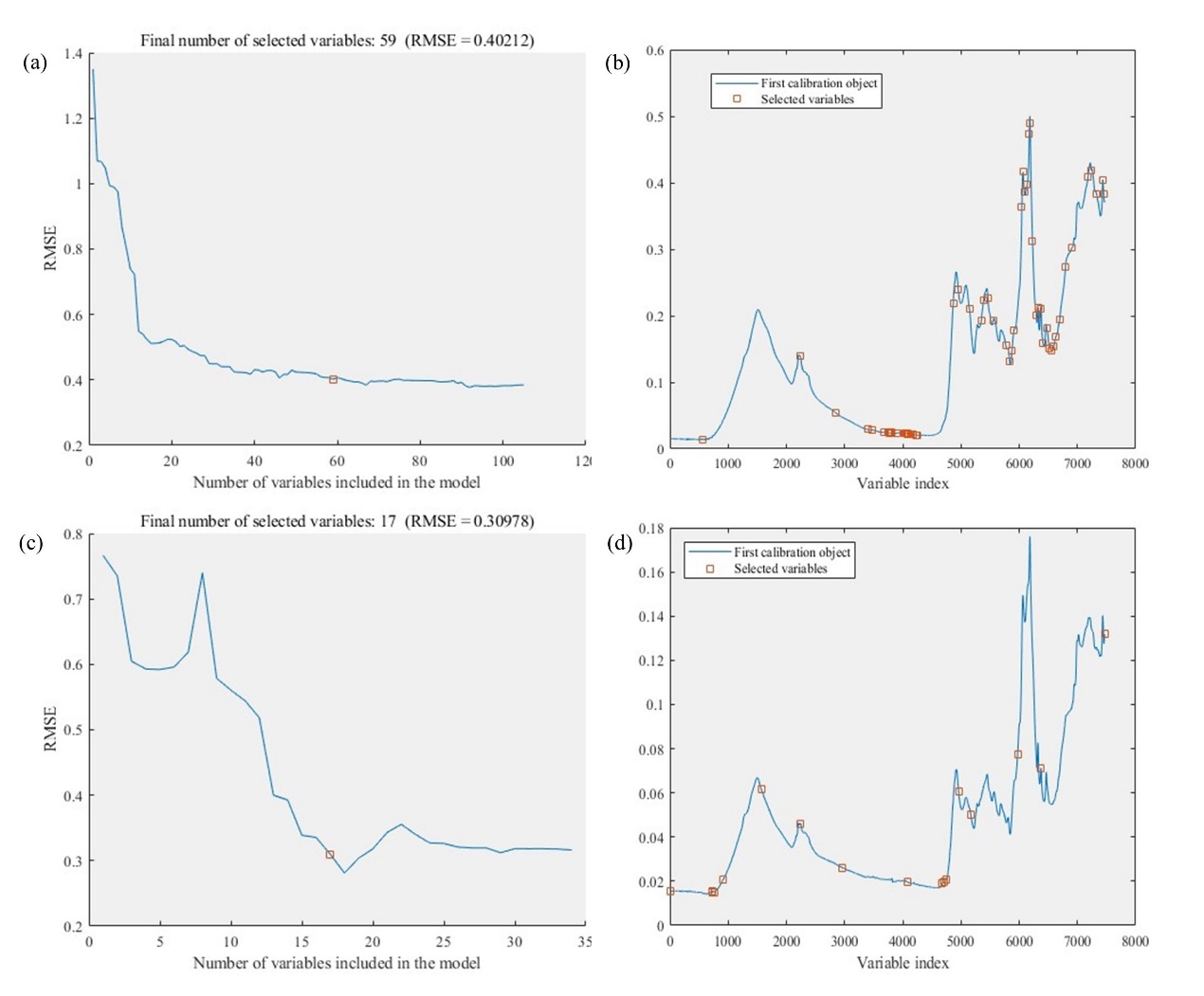


**Fig.S2.** SPA model selection process for feature variables. (a) and (b): Boletus species identification model, (c) and (d): Boletus origin identification model. Where (a) and (c) are the final number of variables selected based on smaller RMSE values and fewer number of variables, and (b) and (d) are the results of feature variable selections.

**
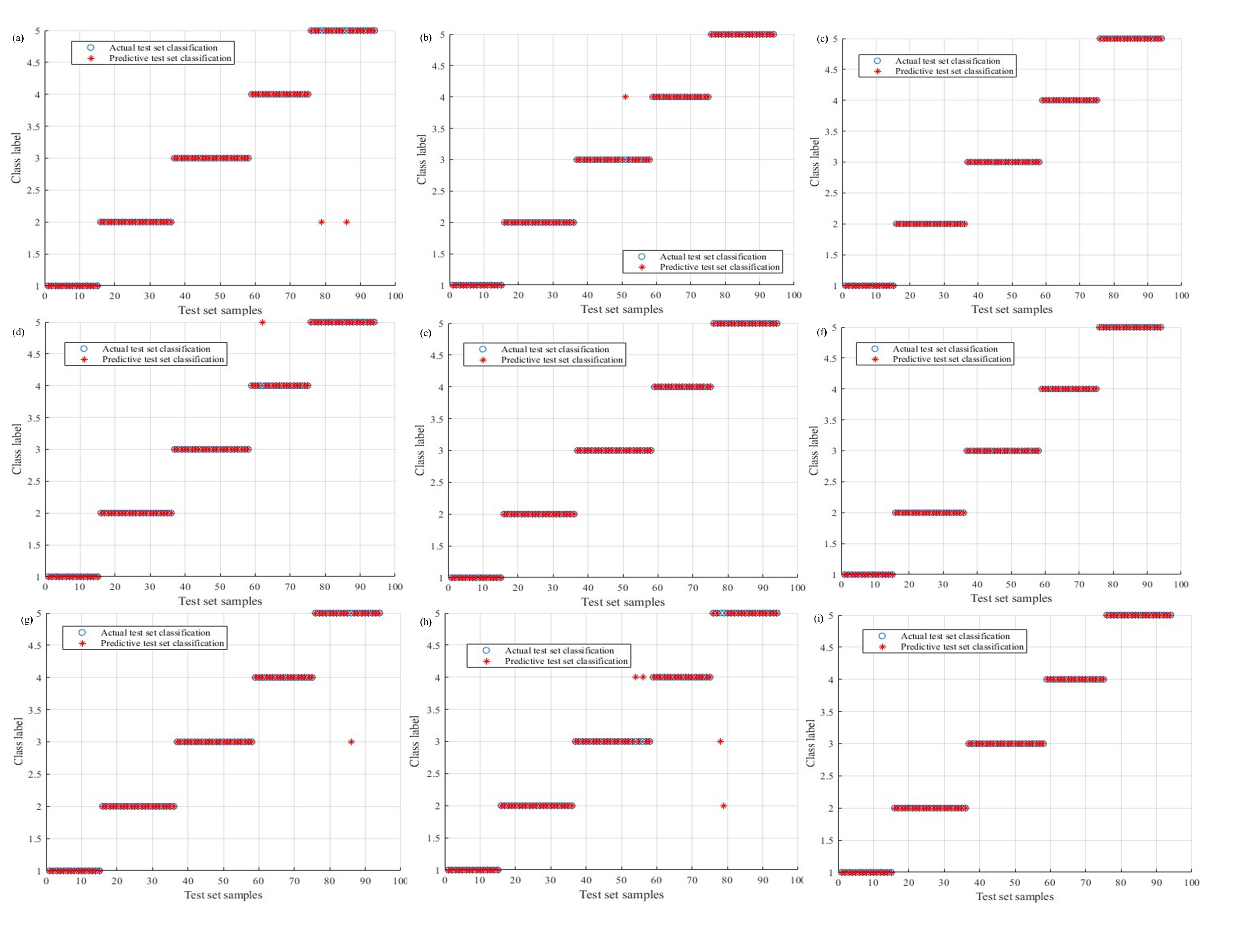
**

**Fig.S3.** Classification results of LIBSVM models of the test and training sets of boletus species under different feature variable selection methods. (a) is the Raw-LIBSVM model. (b) is the manual wavenumbers-LIBSVM model. (c) is the manual wave bands-LIBSVM model. (d) is the fingerprint region-LIBSVM model. (e) is the SD-LIBSVM model. (f) is the VIP(＞1)-LIBSVM model. (g) is the VIP(＞1.5)-LIBSVM model. (h) is the CARS-LIBSVM model. (i) is the SPA-LIBSVM model.

**
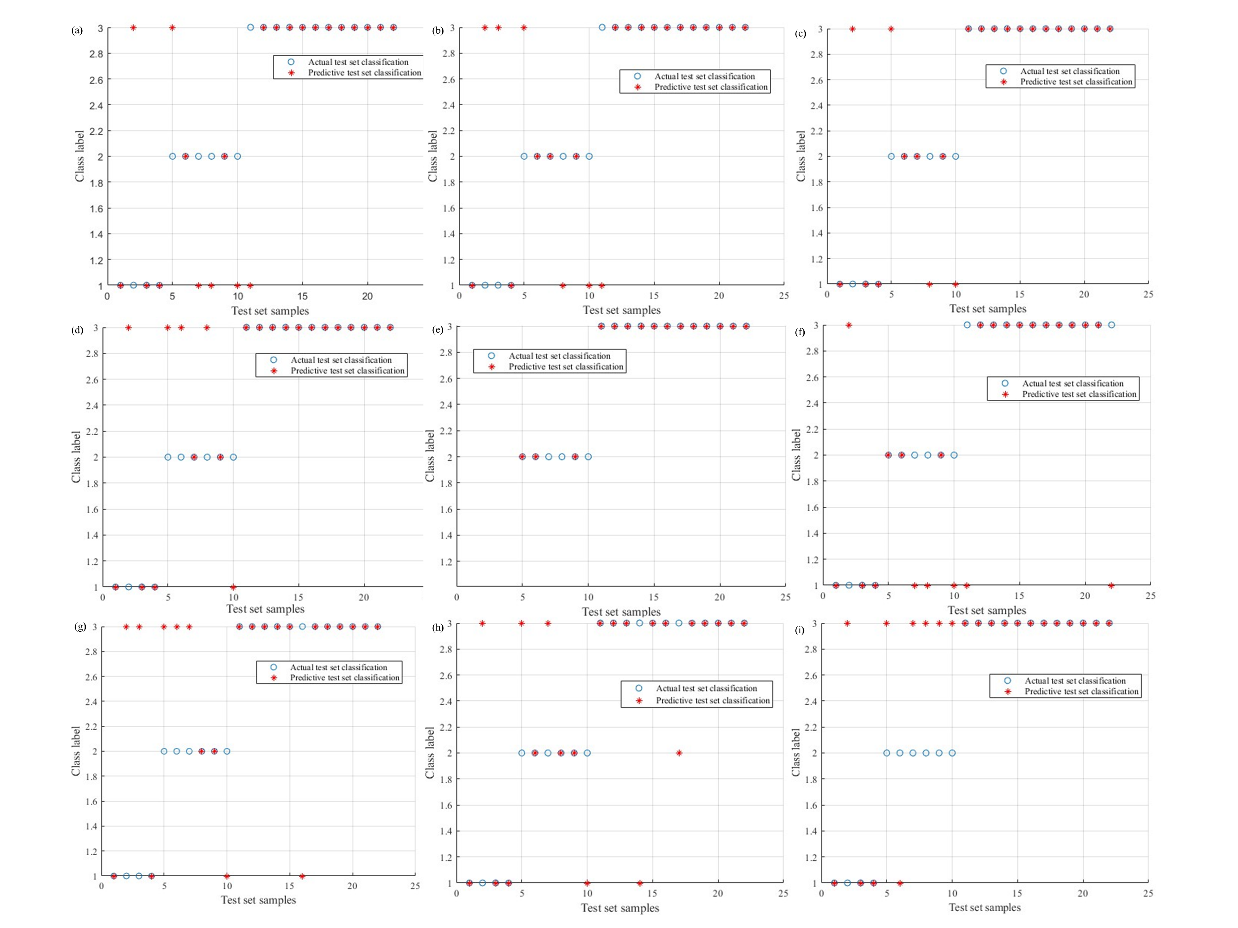
**

**Fig.S4.** Classification results of LIBSVM models of the test and training sets of boletus origins under different feature variable selection methods. (a) is the Raw-LIBSVM model. (b) is the manual wavenumbers-LIBSVM model. (c) is the manual wave bands-LIBSVM model. (d) is the fingerprint region-LIBSVM model. (e) is the SD-LIBSVM model. (f) is the VIP(＞1)-LIBSVM model. (g) is the VIP(＞1.5)-LIBSVM model. (h) is the CARS-LIBSVM model. (i) is the SPA-LIBSVM model.
